# Correlation between Genomic Variants and Worldwide Epidemiology of Prostate Cancer

**DOI:** 10.3390/genes13061039

**Published:** 2022-06-10

**Authors:** Giovana Miranda Vieira, Laura Patrícia Albarello Gellen, Diana Feio da Veiga Borges Leal, Lucas Favacho Pastana, Lui Wallacy Morikawa Souza Vinagre, Vitória Teixeira Aquino, Marianne Rodrigues Fernandes, Paulo Pimentel de Assumpção, Rommel Mario Rodríguez Burbano, Sidney Emanuel Batista dos Santos, Ney Pereira Carneiro dos Santos

**Affiliations:** 1 Research Center of Oncology, Federal University of Pará Belém, Belém 66073-000, Brazil; giovanamirandav@gmail.com (G.M.V.); laura.patricia.agellen@hotmail.com (L.P.A.G.); dianafeio@hotmail.com (D.F.d.V.B.L.); lucas.pastana@ics.ufpa.br (L.F.P.); luivinagre@gmail.com (L.W.M.S.V.); vit.teixeiraa@gmail.com (V.T.A.); assumpcaopp@gmail.com (P.P.d.A.); rommelburbano@gmail.com (R.M.R.B.); sidneysantos@ufpa.br (S.E.B.d.S.); npcsantos.ufpa@gmail.com (N.P.C.d.S.); 2 Ophir Loyola Hospital, Belém 66063-005, Brazil

**Keywords:** prostate cancer, genetic risk variant, susceptibility, severity, epidemiology, population

## Abstract

Prostate cancer (PCa) incidence and mortality vary across territories and populations. This can be explained by the genetic factor of this disease. This article aims to correlate the epidemiological data, worldwide incidence, and mortality of PCa with single-nucleotide polymorphisms (SNPs) associated with the susceptibility and severity of this neoplasm in different populations. Eighty-four genetic variants associated with prostate cancer susceptibility were selected from the literature through genome association studies (GWAS). Allele frequencies were obtained from the 1000 Genomes Project, and epidemiological data were obtained from Surveillance, Epidemiology, and End Results (SEER). The PCa incidence, mortality rates, and allele frequencies of variants were evaluated by Pearson’s correlation. Our study demonstrated that 12 SNPs (rs2961144, rs1048169, rs7000448, rs4430796, rs2066827, rs12500426, rs6983267, rs11649743, rs2075110, rs114798100, rs855723, and rs2075109) were correlated with epidemiological data in different ethnic groups. Ten SNPs (rs2961144, rs1048169, rs7000448, rs4430796, rs2066827, rs12500426, rs11649743, rs2075110, rs114798100, and rs2075109) were positively correlated with the mortality rate. Seven SNPs (rs1048169, rs2961144, rs7000448, rs4430796, rs2066827, rs12500426, and rs114798100) were positively correlated with incidence. Positive correlations of incidence and mortality rates were more frequent in the African population. The genetic variants investigated here are likely to predispose to PCa and could play a role in its progression and aggressiveness. This genetic study demonstrated here is promising for implementing personalized strategies to screen for prostate cancer in diverse populations.

## 1. Introduction

Prostate cancer (PCa) is the second most common cancer and the fifth leading cause of cancer death among men. In 2020, it was estimated that approximately 1.4 million new cases and 375,000 deaths from prostate cancer occurred worldwide. The etiology of prostate cancer is multifactorial, with some risk factors associated with its development, including advanced age, positive family history, and African ancestry [1].

PCa incidence varies across ethnic groups and geographic variations. There are higher incidence and mortality rates in people of Black African descent worldwide [2]. In the United States, there is variability among different ethnic groups, with the highest incidence and mortality in Black men of African descent. The difference in incidence and mortality can be attributed to the prevalence of multiple prostate cancer genetic risk loci across ethnic groups [3,4].

Recent studies of epidemiology suggest that PCa is a highly heritable disease and suggests a strong causal association between genetic factors and the development of prostate cancer. A twin study revealed that 57% of prostate cancer individuals had a family link to prostate cancer [5]. Recent studies indicate that more than 100 well-recognized SNPs have been found to be associated with prostate cancer and constitute a major risk factor in the development of prostate cancer [6].

GWAS have changed the field of cancer genetics in the last decade. GWAS have improved the understanding of the key genes and gene variants involved in molecular and biological mechanisms that involve PCa etiology. Although PCa incidence and mortality rates are higher in African descent, most large-scale GWAS have been conducted in the European population [7].

This article aims to correlate epidemiological data on the incidence and mortality of prostate cancer worldwide with frequencies of important SNPs in studies of GWAS associated with the susceptibility and severity of this neoplasm in different populations.

## 2. Materials and Methods

### 2.1. SNP Determination

**Figure 1:**
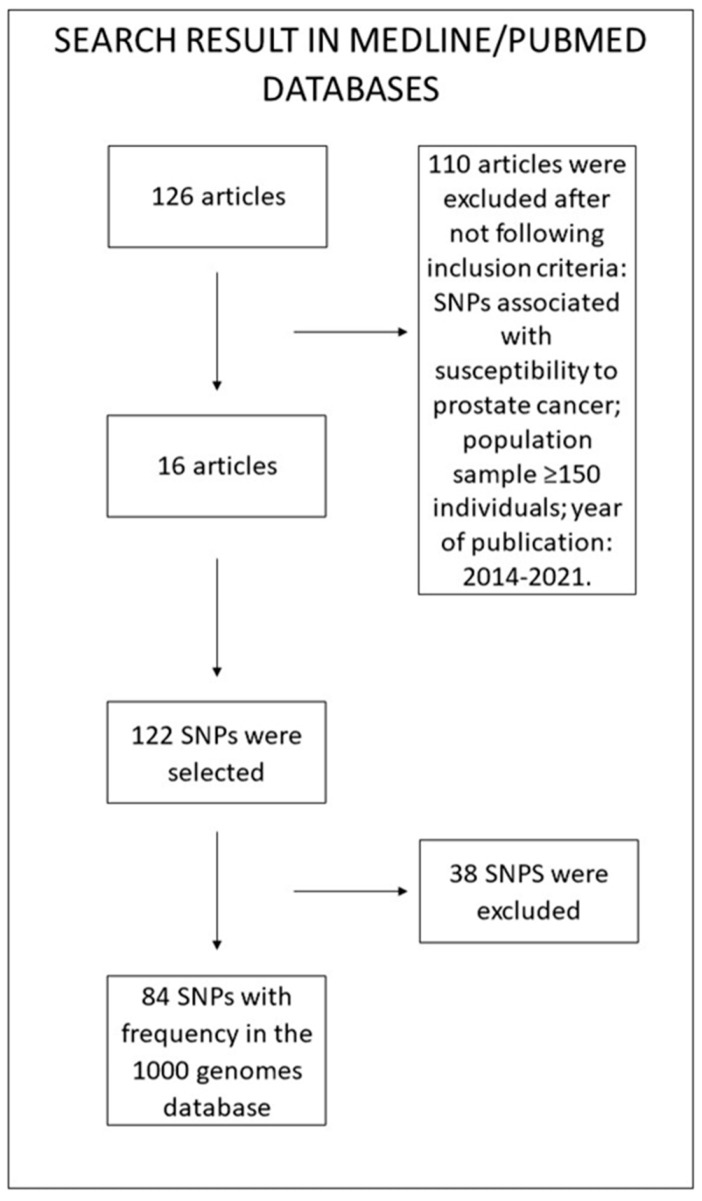
SNP selection flowchart.

We searched online Medline/PubMed databases for articles published in English using several key terms relating to prostate cancer and susceptibility. The key terms were prostate cancer, GWAS, risk, susceptibility, and population, resulting in 126 articles. There were 16 articles after using the following inclusion criteria: studies on SNPs associated with susceptibility to prostate cancer, with a population sample greater than 150 individuals, and in the period from 2014 to 2021. The result was 122 polymorphisms related to prostate cancer susceptibility. Among these, we selected 84 SNPs that had their frequency detected in the 1000 Genomes database, and 38 were excluded from the study because they were not detected in this database (Figure 1).

### 2.2. Epidemiological and Genetic Data

The incidence and mortality rates of PCa were obtained from Surveillance, Epidemiology, and End Results (SEER)-Medicare [3] in the United States. In this database, the ethnicities reported were White non-Hispanic, White Hispanic, Black, Asian/Pacific Islander, Amerindian/Alaska Native, and Hispanic. In the 1000 Genomes Project, the ethnicities reported were from: Europe (EUR), Africa (AFR), East Asia (EAS), South Asia (SAS), and the Americas (AMR). In our study, we classified the populations from SEER into 6 groups. The EUR group was composed of White non-Hispanic and White Hispanic populations. The AFR group was composed of the Black population. The EAS and SAS groups were averages of the Asian/Pacific Islander population. The AMR group was composed of Amerindian/Alaska Native and Hispanic groups. Gene allele frequencies were assessed from phase 3 data from the 1000 Genomes Project for Africa, East Asia, Europe, South Asia, and the Americas.

### 2.3. Statistical Analysis

The prostate incidence, mortality rates, and allele frequencies of the variants were evaluated by Pearson’s correlation. The data were evaluated with previously described groups, using the “cor. test” function of the “stats” package of the R programming language. After this procedure, the values of r, r^2^, *p*-value, and 95% CI were obtained. All plots were created using the “ggplot2” graphics package. A *p*-value less than 0.05 (*p* ≤ 0.05) was statistically significant.

## 3. Results

**Figure 2:**
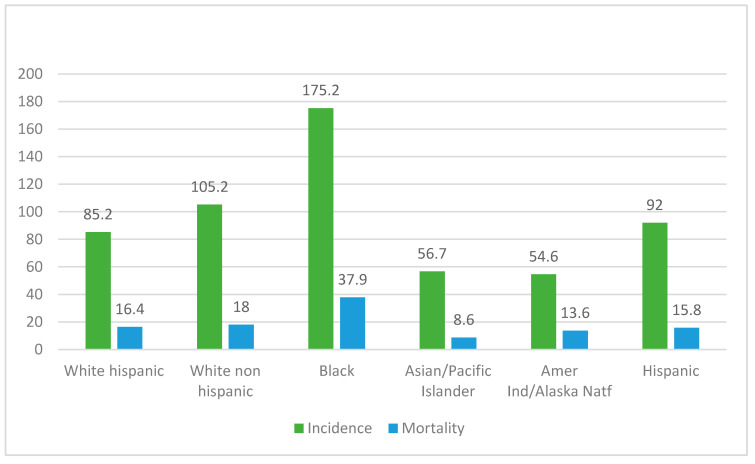
Incidence and mortality rates per 100.000 habitants from SEER prostate cancer registries from 2013 to 2017 in different populations.

The incidence rate of prostate cancer was 175.2 per 100.000 men in the Black population, followed by White non-Hispanic (105.2), Hispanic (92), White Hispanic (85.2), Asian/Pacific Islander (56.7), and Amerindian/Alaska Native populations (54.6). The mortality rate of prostate cancer was also higher in the Black population at 37.9 per 1000.000, followed by White non-Hispanic (18), White Hispanic (16.4), Hispanic (15.8), Amerindian/Alaska Native (13.6), and Asian/Pacific Islander (8.6) (Figure 2).

**Figure 3:**
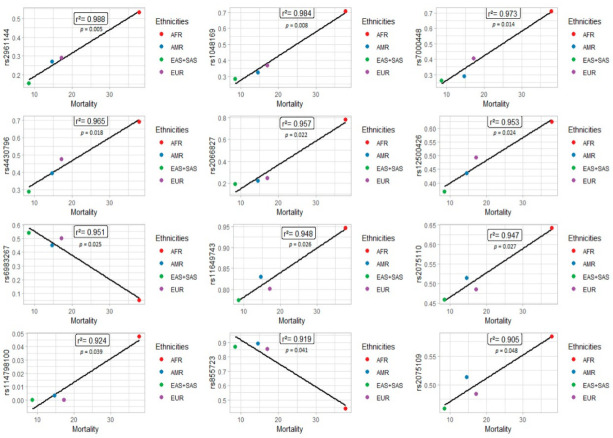
SNPs associated with prostate cancer mortality in different populations.

A total of 84 SNPS (Supplementary Tables S1–S3) were selected from the literature, as described in Figure 1. Among those polymorphisms, twelve SNPs (rs2961144, rs1048169, rs7000448, rs4430796, rs2066827, rs12500426, rs6983267, rs11649743, rs2075110, rs114798100, rs855723, and rs2075109) were significant in Pearson’s correlation analysis (Table 1 and Table 2). Among these 12, 10 (rs2961144, rs1048169, rs7000448, rs4430796, rs2066827, rs12500426, rs11649743, rs2075110, rs114798100, and rs2075109) were positively correlated with the mortality rate (Figure 3).

The higher the frequency of the variant allele of these polymorphisms in populations, the higher the estimated mortality rates. All ten variants (rs2961144, rs1048169, rs7000448, rs4430796, rs2066827, rs12500426, rs11649743, rs2075110, rs114798100, and rs2075109) were more frequent in the African population, which demonstrates the greater aggressiveness of prostate cancer in the African population.

Nevertheless, two polymorphisms (rs6983267 and rs855723) were inversely correlated with the mortality rate. Consequently, the higher the frequency of the variant allele, the lower the estimated mortality rate. Both variants were more frequent in the East Asian population, possibly because East Asian ancestry may be a protective factor against prostate cancer.

Only twelve genetic variants were significantly correlated with worldwide prostate cancer mortality data. More information about the location, impact, relative risk, and allele frequencies of the variants are presented in Table 1.

**Figure 4:**
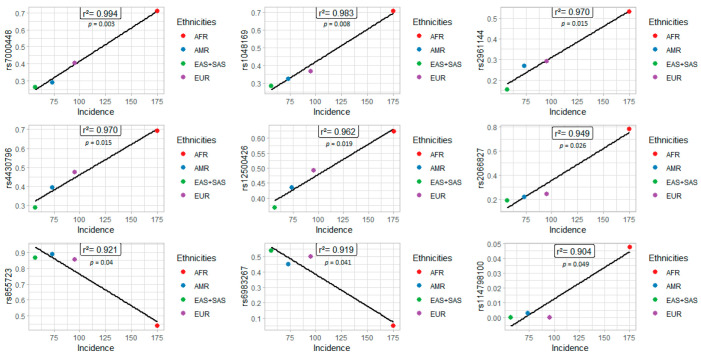
SNPs associated with prostate cancer incidence in different populations.

Nine variants (rs7000448, rs1048169, rs2961144, rs4430796, rs12500426, rs2066827, rs6983267, rs855723, and rs114798100) had a statistically significant correlation with the incidence rate (Figure 4). Among nine SNPs, seven variants (rs7000448, rs1048169, rs2961144, rs4430796, rs12500426, rs2066827, and rs114798100) were positively correlated with the incidence rate. The higher the frequency of the variant, the greater the number of new cases of prostate cancer. Seven polymorphisms (rs1048169, rs2961144, rs7000448, rs4430796, rs2066827, rs12500426, and rs114798100) were more frequent in the African population, which indicates high susceptibility to prostate cancer in Africans.

However, two polymorphisms (rs6983267 and rs855723) were inversely correlated with the incidence rate, so the higher the frequency of the variant allele, the lower the incidence rate. Both variants also had a lower mortality rate, which reinforces the protective action of the allele against prostate cancer in the East Asian population.

For nine variants, there were significant correlations between variations in allelic frequencies and the incidence of prostate cancer in different populations (Table 2). More information about the genes and their respective IDs, the location of these variants, clinical impact, and the relative risk are presented in Table 2.

## 4. Discussion

Prostate cancer incidence and mortality rates vary significantly by ethnicity, showing a wide fluctuation in epidemiological rates and evidencing a higher incidence and worse prognosis in men of African descent [8]. Our results showed that populations of African origin had higher incidence and mortality rates of the disease, and, contrary to populations of Asian origin, they obtained better results related to susceptibility and mortality.

This study correlated epidemiological data on the incidence and mortality of prostate cancer worldwide with frequencies of important SNPs in studies of GWA associated with the susceptibility and severity of this neoplasm in different populations. Our results indicated correlations for 12 genetic variants (rs2961144, rs7000448, rs4430796, rs2066827, rs12500426, rs6983267, rs1649743, rs1649743, rs20751010, rs2075109, rs114798100, rs114798100, and rs855723) in 10 genes (*HNF1B, EGFR, CCAT2, WNT, PCAT2, PDLIM5, CDKN1B, CASC8, HAUS6i,* and *OR2A5*) with incidence and mortality rates. Our results show that the variants related to high incidence and mortality are more frequent in the African population and less frequent in the Asian population.

*Hepatocyte Nuclear Factor 1 β (HNF1B)* is a transcription factor-2 that regulates metabolic pathways and genes important for human embryonic development [9]. The gene is essential for the embryonic formation of the genital tract, pancreas, liver, biliary tract, and gastrointestinal system [10]. In our studies, SNPs rs4430796 and rs11649743 were concomitantly associated with a higher incidence and mortality of prostate cancer. Both variants have been linked to an increased risk of prostate cancer in several studies [11,12].

The *Epidermal Growth Factor Receptor* (*EGFR*) gene acts in the regulation of proliferation, differentiation, division, survival, and cancer development [13]. In 2004, Shuch et al. reported increased gene expression in African Americans with prostate cancer. In our study, the SNPs in *EGFR* (rs2075110 and rs2075109) were correlated with higher mortality and susceptibility to prostate cancer. Both variants were more frequent in Africans.

The *Colon cancer-associated transcript 2* (*CCAT2*) gene was first associated with colon cancer; however, it is currently associated with several types of cancer, including prostate cancer [14]. Peng He et al., when analyzing 18 patients with prostate cancer, found a high expression of *CCAT2*, indicating a role of this gene in the pathogenesis and progression of the disease [15]. In our study, the rs6983267 SNP of the *CCAT2* gene was inversely related to mortality and incidence rates. The higher the frequency of this marker, the lower the mortality and incidence rates. This SNP was more frequent in the Asian population and less frequent in Africans.

The *Wingless-type (WNT)* proteins are involved in bone metabolism, affecting several diseases [16]. The *WNT* gene is related to PCa bone metastasis and is found at high levels in the advanced stages of this disease [17]. In our study, the variant rs855723 allele was inversely correlated with incidence and mortality. Wang et al. found that rs855723 was associated with lower expression of *WNT1*, and this corroborates our finding on mortality.

The *PDLIM5* gene is a protein that binds to several proteins, such as PKC, PKA, PKD, and AMPK, and is important in signal modulation pathways [18]. In our study, rs12500426 was associated with higher mortality and incidence, being more frequent in Africans.

The *CDKN1B* gene is responsible for encoding a protein that inhibits a cyclin-dependent kinase that plays an important role in the cell cycle [19]. Other studies, such as that by Farashi et al. (2018), have demonstrated the possible association of rs2066827 with the risk of developing PCa, resulting from a missense mutation (V109G) in the *CDKN1B* gene that contributes to prostate cancer tumorigenesis through the deregulation of cell cycle checkpoints [20]; these data corroborate the findings in our study, which demonstrate a positive correlation between the increase in the frequency of this SNP and the increase in the incidence of cases.

The *CASC8* gene is responsible for expressing a lncRNA (long non-coding RNA) that plays an important role in the regulation of MYC, which is related to susceptibility to various cancers, such as breast, colorectal, and prostate cancers [21,22]. Our findings demonstrate a directly proportional correlation of rs7000448 in this gene with the mortality and incidence of prostate cancer, especially in the African population. These findings are in agreement with international studies, which demonstrated that *CASC8* and rs7000448 are mainly related to PCa, such as in the meta-analysis carried out by Tong (2020). This polymorphism was related to the increase in cases in Caucasians and Africans [23,24,25].

*HAUS6* is part of the group of augmin complex subunits, which play an important role in the recruitment and amplification of microtubule molecules during cell division [26]. Our analysis demonstrated a significant correlation of rs1048169 in this gene with incidence and mortality, which were higher in the African population. There are few studies correlating this polymorphism with the risk of developing PCa; however, Schumacher and colleagues pointed out a possible role of this variant in the risk of developing the neoplasm [19].

*OR2A5* is a gene responsible for expressing hormone receptors and neurotransmitters. Lin and colleagues found results in agreement with our study, where *OR2A5* was also related to the risk of developing prostate cancer [27]. In our findings, rs2961144 showed a positive correlation with the mortality rate mainly in African populations.

The SNP rs114798100 in the *PCAT2* gene is described as an important risk marker for prostate cancer development. It is considered a rare variant and found only in African American populations [28]. In our study, this polymorphism was also related to the risk of developing prostate cancer.

This study had limitations regarding ethnic group information, such as the Amerindians. The study also had a lack of homogeneity in the socioeconomic levels in studies. Despite these limitations, this study contributes important findings to the literature, and it may help to choose genetic markers that are more globally homogeneous in relation to the prognosis and predisposition to prostate cancer.

These genetic variants likely predispose to PCa and may play a role in PCa progression and aggressiveness. The SNPs studied, which were positively correlated with incidence and mortality rates, were more frequent in the African population. This genetic study holds promise for implementing personalized strategies for prostate cancer screening in diverse populations.

## 5. Conclusions

Our study demonstrated that 12 SNPs (rs2961144, rs1048169, rs7000448, rs4430796, rs2066827, rs12500426, rs6983267, rs11649743, rs2075110, rs114798100, rs855723, and rs2075109) were correlated with epidemiological data in different ethnic groups. Ten SNPs (rs2961144, rs1048169, rs7000448, rs4430796, rs2066827, rs12500426, rs11649743, rs2075110, rs114798100, and rs2075109) were positively correlated with the mortality rate. Seven polymorphisms (rs1048169, rs2961144, rs7000448, rs4430796, rs2066827, rs12500426, and rs114798100) were positively correlated with incidence. In contrast, two polymorphisms (rs6983267 and rs855723) were negatively correlated with mortality and incidence rates.

## Figures and Tables

**Table 1:** SNPs significantly correlated with PCa mortality in different populations.

Gene	SNP ID	Gene Consequence	Alleles	Ancestral	Placement	Clinical Impact	MAF	Frequency from the 1000 Genomes Project				
								AFR	EAS	SAS	EUR	AMR
*OR2A5*	rs2961144	Missense Variant	A/G	A	GRCh38.p13 chr 7	Not Reported	0.30	0.5318	0.0079	0.298	0.2903	0.269
*HAUS6*	rs1048169	3’ UTR Variant	T/C/G	C	GRCh38.p13 chr 9	Not Reported	0.42	0.7073	0.2073	0.36	0.3668	0.323
*CASC8*	rs7000448	Intron Variant	C/T	T	GRCh38.p13 chr 8	Not Reported	0.41	0.711	0.2659	0.258	0.4046	0.29
*HNF1B*	rs4430796	Intron Variant	A/C/G/T	G	GRCh38.p13 chr 17	Not Reported	0.49	0.6906	0.2768	0.297	0.4751	0.395
*CDKN1B*	rs2066827	Missense Variant	T/A/C/G	T	GRCh38.p13 chr 12	Benign	0.36	0.7837	0.0556	0.319	0.2425	0.218
*PDL1M5*	rs12500426	Intron Variant	A/C	C	GRCh38.p13 chr 4	Not Reported	0.48	0.6225	0.4931	0.241	0.4931	0.435
*CCAT2*	rs6983267	Non-Coding Transcript Variant	G/T	G	GRCh38.p13 chr 8	Not Reported	0.39	0.041	0.0477	0.6121	0.468	0.5010
*HNF1B*	rs11649743	Intron Variant	A/G	G	GRCh38.p13 chr 17	Not Reported	0.37	0.9470	0.6617	0.885	0.8012	0.829
*EGFR*	rs2075110	Intron Variant	C/G/T	T	GRCh38.p13 chr 7	Not Reported	0.48	0.643	0.3522	0.565	0.565	0.514
*PCAT2*	rs114798100	Intron Variant	A/G	A	GRCh38.p13 chr 8	Not Reported	0.01	0.0477	0	0	0	0.003
*WNT1*	rs855723	Regulatory Region Variant	G/A/C/T	G	GRCh38.p13 chr 12	Not Reported	0.25	0.4365	0.996	0.737	0.8549	0.889
*EGFR*	rs2075109	Intron Variant	T/C	C	GRCh38.p13 chr 7	Not Reported	0.50	0.584	0.3522	0.564	0.4841	0.513

**Table 2:** SNPs significantly correlated with PCa incidence in different populations.

Gene	SNP ID	Gene consequence	Alleles	Ancestral	Placement	Clinical Impact	MAF	Frequency from the 1000 Genomes Project				
								AFR	EAS	SAS	EUR	AMR
*CASC8*	rs7000448	Intron Variant	C/T	T	GRCh38.p13 chr 8	Not Reported	0.41	0.711	0.2659	0.258	0.4046	0.29
*HAUS6*	rs1048169	3 Prime UTR Variant	T/C/G	C	GRCh38.p13 chr 9	Not Reported	0.42	0.7073	0.2073	0.36	0.3668	0.323
*NF1B*	rs4430796	Intron Variant	A/C/G/T	G	GRCh38.p13 chr 17	Not Reported	0.49	0.6906	0.2768	0.297	0.4751	0.395
*OR2A5*	rs2961144	Missense Variant	A/G	A	GRCh38.p13 chr 7	Not Reported	0.30	0.5318	0.0079	0.298	0.903	0.269
*PDL1M5*	rs12500426	Intron Variant	A/C	C	GRCh38.p13 chr 4	Not Reported	0.48	0.6225	0.4931	0.241	0.4931	0.435
*CDKN1B*	rs2066827	Missense Variant	T/A/C/G	T	GRCh38.p13 chr 12	Benign	0.36	0.7837	0.0556	0.319	0.2425	0.218
*WNT1*	rs855723	Regulatory Region Variant	G/A/C/T	G	GRCh38.p13 chr 12	Not Reported	0.25	0.4365	0.996	0.737	0.8549	0.889
*CCAT2*	rs6983267	Non-Coding Transcript Exon Variant	G/T	G	GRCh38.p13 chr 8	Not Reported	0.39	0.0477	0.6121	0.468	0.5010	0.450
*PCAT2*	rs114798100	Intron Variant	A/G	A	GRCh38.p13 chr 8	Not Reported	0.01	0.0477	0	0	0	0.003

## Data Availability

All relevant data will be shared as Supporting Information files if the manuscript is accepted for publication.

## Supplementary Materials

The following supporting information can be downloaded at: https://www.mdpi.com/article/10.3390/genes13061039/s1. Table S1: SNPs correlated with PCa mortality in different populations; Table S2: SNPs correlated with PCa incidence in different populations. Table S3: SNPs correlated with PCa mortality and incidence in different populations.
