# Supplementary material for: Correlation between Genomic Variants and Worldwide Epidemiology of Prostate Cancer
Source: Genes (Basel). 2022 Jun 10;13(6):1039. doi: 10.3390/genes13061039 (PMC9222668; doi:10.3390/genes13061039)
Supplement: Supplementary file 1 [file genes-13-01039-s001.zip › genes-1712162-supplementary.pdf]

Supplementary Table S1. SNPs correlated with PCa mortality in different populations.

SNP	r	r2	*p* Value	IC95%_Min	IC95%_Max
**rs2961144**	0.994	0.988	0.006	0.7287	0.999876
**rs1048169**	0.992	0.984	0.008	0.661132	0.999839
**rs7000448**	0.986	0.973	0.014	0.48113	0.999724
**rs4430796**	0.982	0.965	0.018	0.379361	0.999646
**rs2066827**	0.978	0.957	0.022	0.281966	0.999559
**rs12500426**	0.976	0.953	0.024	0.242836	0.99952
**rs6983267**	-0.975	0.951	0.025	-0.9995	-0.2261
**rs11649743**	0.974	0.948	0.026	0.196545	0.999471
**rs2075110**	0.973	0.947	0.027	0.182585	0.999456
**rs114798100**	0.961	0.924	0.039	0.00064	0.999214
**rs855723**	-0.959	0.919	0.041	-0.99916	0.029871
**rs2075109**	0.952	0.905	0.048	-0.11206	0.999014
**rs12718946**	0.947	0.896	0.053	-0.15932	0.998915
**rs9364554**	-0.933	0.871	0.067	-0.99863	0.271563
**rs9469899**	0.933	0.871	0.067	-0.2705	0.99863
**rs12665339**	-0.92	0.846	0.08	-0.99834	0.357279
**rs527510716**	-0.918	0.843	0.082	-0.99831	0.365712
**rs7975232**	0.913	0.834	0.087	-0.39148	0.998201
**rs4976790**	0.907	0.823	0.093	-0.42055	0.998071
**rs817826**	-0.902	0.814	0.098	-0.99797	0.442269
**rs7584330**	0.902	0.813	0.098	-0.44547	0.99795
**rs61088131**	0.895	0.801	0.105	-0.47191	0.997808
**rs11672691**	0.888	0.788	0.112	-0.49973	0.997642
**rs878987**	0.887	0.787	0.113	-0.50258	0.997624
**rs6983561**	0.867	0.752	0.133	-0.56416	0.997178
**rs16901979**	0.863	0.744	0.137	-0.57585	0.997079
**rs7094871**	0.859	0.738	0.141	-0.58451	0.997002
**rs34579442**	-0.855	0.732	0.145	-0.99691	0.594398
**rs2680708**	0.843	0.711	0.157	-0.6219	0.996628
**rs1182**	-0.84	0.705	0.16	-0.99655	0.629012
**rs11629412**	-0.837	0.701	0.163	-0.99648	0.634528
**rs73199732**	0.832	0.693	0.168	-0.64359	0.996375
**rs1859962**	0.83	0.69	0.17	-0.64707	0.996332
**rs17621345**	-0.83	0.688	0.17	-0.99631	0.648639
**rs55851920**	0.83	0.689	0.17	-0.64739	0.996328
**rs1465618**	0.828	0.685	0.172	-0.65219	0.996266
**rs56197129**	0.828	0.685	0.172	-0.65245	0.996263
**rs6782221**	0.824	0.678	0.176	-0.65943	0.99617
**rs6795465**	0.817	0.667	0.183	-0.67095	0.996009
**rs56413159**	0.802	0.643	0.198	-0.69394	0.995651
**rs12791447**	-0.791	0.625	0.209	-0.99537	0.709864
**rs34925593**	-0.784	0.615	0.216	-0.99521	0.718358

**rs6944695**	0.784	0.614	0.216	-0.71882	0.995198
**rs6501455**	-0.766	0.587	0.234	-0.99476	0.739153
**rs2075111**	0.749	0.561	0.251	-0.75724	0.994316
**rs1512268**	-0.745	0.554	0.255	-0.99421	0.76133
**rs1935581**	0.741	0.55	0.259	-0.76426	0.994124
**rs2270247**	0.671	0.45	0.329	-0.81679	0.992221
**rs28756990**	0.664	0.441	0.336	-0.82086	0.992028
**rs10486567**	-0.663	0.439	0.337	-0.99199	0.821722
**rs2072454**	0.642	0.413	0.358	-0.83302	0.991393
**rs730437**	0.642	0.412	0.358	-0.83327	0.991379
**rs10993994**	-0.63	0.396	0.37	-0.99102	0.839469
**rs721048**	-0.628	0.395	0.372	-0.99098	0.840067
**rs74702681**	-0.621	0.385	0.379	-0.99076	0.843634
**rs7616437**	0.593	0.351	0.407	-0.85594	0.989906
**rs17321482**	-0.57	0.325	0.43	-0.98921	0.86471
**rs2928679**	0.566	0.32	0.434	-0.86651	0.98905
**rs17021918**	-0.524	0.275	0.476	-0.98769	0.880479
**rs6465657**	-0.514	0.264	0.486	-0.98734	0.883564
**rs9632117**	-0.501	0.251	0.499	-0.98689	0.88735
**rs28441558**	0.48	0.23	0.52	-0.8931	0.986151
**rs10793821**	0.422	0.178	0.578	-0.90692	0.983996
**rs182314334**	-0.387	0.15	0.613	-0.98262	0.914017
**rs59308963**	0.353	0.124	0.647	-0.92039	0.981183
**rs11691517**	0.327	0.107	0.673	-0.92475	0.980059
**rs12621278**	-0.319	0.102	0.681	-0.97971	0.925999
**rs28607662**	-0.301	0.091	0.699	-0.9789	0.928785
**rs1800057**	-0.279	0.078	0.721	-0.97787	0.932006
**rs2735839**	-0.258	0.066	0.742	-0.97684	0.93497
**rs12785905**	-0.248	0.062	0.752	-0.97638	0.93622
**rs33984059**	-0.19	0.036	0.81	-0.97336	0.943328
**rs7295014**	0.183	0.034	0.817	-0.94411	0.972979
**rs7679673**	0.175	0.031	0.825	-0.94503	0.97252
**rs58262369**	0.17	0.029	0.83	-0.94557	0.972241
**rs4962416**	-0.127	0.016	0.873	-0.96974	0.950028
**rs11290954**	-0.121	0.015	0.879	-0.96939	0.95059
**rs1004030**	0.117	0.014	0.883	-0.95099	0.969138
**rs1283104**	-0.085	0.007	0.915	-0.96706	0.954054
**rs76551843**	0.07	0.005	0.93	-0.9554	0.966062
**rs2277283**	-0.046	0.002	0.954	-0.96445	0.957417
**rs72725879**	0.021	0	0.979	-0.95945	0.962658
**rs11452686**	-0.017	0	0.983	-0.96239	0.959743
**rs11666569**	-0.005	0	0.995	-0.96147	0.960703

Supplementary Table S2. SNPs correlated with PCa incidence in different populations.

SNP	r	r2	*p* value	IC95%_Min	IC95%_Max
**rs7000448**	0.997	0.994	0.003	0.860293	0.999941
**rs1048169**	0.992	0.983	0.008	0.648262	0.999832
**rs4430796**	0.985	0.97	0.015	0.445065	0.999698
**rs2961144**	0.985	0.97	0.015	0.440592	0.999694
**rs12500426**	0.981	0.962	0.019	0.345781	0.999617
**rs2066827**	0.974	0.949	0.026	0.209389	0.999485
**rs855723**	-0.96	0.921	0.04	-0.99919	0.016841
**rs6983267**	-0.959	0.919	0.041	-0.99916	0.030113
**rs114798100**	0.951	0.904	0.049	-0.12102	0.998996
**rs11649743**	0.949	0.901	0.051	-0.13664	0.998964
**rs2075110**	0.949	0.9	0.051	-0.13872	0.998959
**rs7975232**	0.945	0.893	0.055	-0.1746	0.99888
**rs12665339**	-0.943	0.89	0.057	-0.99884	0.189805
**rs9469899**	0.938	0.879	0.062	-0.23776	0.998722
**rs527510716**	-0.93	0.865	0.07	-0.99857	0.290586
**rs878987**	0.92	0.847	0.08	-0.35287	0.998355
**rs2075109**	0.919	0.844	0.081	-0.3627	0.998318
**rs12718946**	0.913	0.833	0.087	-0.39325	0.998193
**rs817826**	-0.913	0.833	0.087	-0.99819	0.394257
**rs4976790**	0.902	0.813	0.098	-0.44522	0.997951
**rs9364554**	-0.896	0.803	0.104	-0.99783	0.46834
**rs34579442**	-0.891	0.794	0.109	-0.99772	0.487277
**rs11672691**	0.889	0.789	0.111	-0.49689	0.99766
**rs61088131**	0.887	0.786	0.113	-0.50333	0.997619
**rs7584330**	0.879	0.773	0.121	-0.52893	0.997447
**rs6983561**	0.864	0.747	0.136	-0.57216	0.997111
**rs16901979**	0.861	0.741	0.139	-0.58048	0.997038
**rs1859962**	0.856	0.733	0.144	-0.59244	0.996928
**rs2680708**	0.852	0.726	0.148	-0.60141	0.996841
**rs12791447**	-0.844	0.713	0.156	-0.99666	0.619172
**rs73199732**	0.844	0.712	0.156	-0.62028	0.996646
**rs55851920**	0.844	0.713	0.156	-0.61943	0.996655
**rs56197129**	0.841	0.707	0.159	-0.62628	0.996579
**rs6782221**	0.838	0.702	0.162	-0.6328	0.996505
**rs6795465**	0.83	0.688	0.17	-0.6489	0.996309
**rs7094871**	0.827	0.684	0.173	-0.65344	0.99625
**rs56413159**	0.817	0.668	0.183	-0.6707	0.996013
**rs17621345**	-0.814	0.663	0.186	-0.99595	0.674946
**rs1182**	-0.806	0.65	0.194	-0.99575	0.687856
**rs1465618**	0.805	0.648	0.195	-0.68925	0.995729
**rs11629412**	-0.788	0.621	0.212	-0.99531	0.713285

**rs34925593**	-0.78	0.609	0.22	-0.99512	0.722824
**rs6501455**	-0.739	0.546	0.261	-0.99407	0.76632
**rs1512268**	-0.735	0.54	0.265	-0.99396	0.769958
**rs6944695**	0.732	0.536	0.268	-0.77242	0.993886
**rs2075111**	0.695	0.484	0.305	-0.8011	0.992895
**rs10486567**	-0.692	0.479	0.308	-0.99281	0.803247
**rs1935581**	0.691	0.477	0.309	-0.80434	0.992765
**rs28756990**	0.672	0.451	0.328	-0.81655	0.992233
**rs721048**	-0.644	0.415	0.356	-0.99145	0.831938
**rs10993994**	-0.636	0.405	0.364	-0.99121	0.836151
**rs2270247**	0.629	0.396	0.371	-0.83955	0.991012
**rs7616437**	0.621	0.385	0.379	-0.84364	0.990758
**rs2072454**	0.6	0.36	0.4	-0.85281	0.990137
**rs730437**	0.598	0.357	0.402	-0.85385	0.990061
**rs17321482**	-0.595	0.354	0.405	-0.98996	0.855175
**rs74702681**	-0.552	0.305	0.448	-0.98862	0.871246
**rs2928679**	0.521	0.271	0.479	-0.8815	0.987575
**rs9632117**	-0.495	0.245	0.505	-0.98668	0.889046
**rs6465657**	-0.489	0.239	0.511	-0.98648	0.890602
**rs28441558**	0.488	0.238	0.512	-0.89096	0.986437
**rs17021918**	-0.472	0.223	0.528	-0.98587	0.895127
**rs59308963**	0.421	0.177	0.579	-0.90711	0.983963
**rs11691517**	0.406	0.165	0.594	-0.91022	0.983386
**rs182314334**	-0.368	0.135	0.632	-0.98182	0.917692
**rs10793821**	0.335	0.113	0.665	-0.92331	0.980445
**rs12621278**	-0.307	0.094	0.693	-0.9792	0.927792
**rs28607662**	-0.282	0.08	0.718	-0.97803	0.931535
**rs1800057**	-0.26	0.067	0.74	-0.97695	0.934682
**rs2735839**	-0.236	0.056	0.764	-0.97576	0.937802
**rs12785905**	-0.214	0.046	0.786	-0.97465	0.940486
**rs1004030**	0.208	0.043	0.792	-0.94128	0.974301
**rs7295014**	0.195	0.038	0.805	-0.94283	0.973594
**rs1283104**	-0.159	0.025	0.841	-0.9716	0.946792
**rs7679673**	0.157	0.025	0.843	-0.94696	0.971509
**rs33984059**	-0.139	0.019	0.861	-0.97047	0.948799
**rs76551843**	0.132	0.017	0.868	-0.94959	0.97
**rs4962416**	-0.084	0.007	0.916	-0.96699	0.954154
**rs58262369**	0.083	0.007	0.917	-0.95424	0.966929
**rs11666569**	-0.071	0.005	0.929	-0.96616	0.955273
**rs11290954**	-0.053	0.003	0.947	-0.96495	0.956808
**rs72725879**	0.038	0.001	0.962	-0.95807	0.963888
**rs11452686**	-0.036	0.001	0.964	-0.96372	0.958267
**rs2277283**	-0.015	0	0.985	-0.96222	0.959926

Supplementary Table S3. SNPs correlated with PCa mortality and incidence in different populations.

Gene	SNP ID	Alleles	Ancestral	Localization	Location Alelle
EHBP1	**rs721048**	G/A	G	GRCh38.p13 chr 2	NC_000002.12:g.62904596=
THADA	**rs1465618**	T/C	T	GRCh38.p13 chr 2	NC_000002.12:g.43326810=
ITGA6	**rs12621278**	A/G	A	GRCh38.p13 chr 2	NC_000002.12:g.172446825=
PDL1M5	**rs17021918**	C/T	C	GRCh38.p13 chr 4	NC_000004.12:g.94641726=
PDL1M5	**rs12500426**	A/C	A	GRCh38.p13 chr 4	NC_000004.12:g.94593458=
TET2	**rs7679673**	C/A/T	C	GRCh38.p13 chr 4	NC_000004.12:g.105140377=
SLC22A3	**rs9364554**	C/T	C	GRCh38.p13 chr 6	NC_000006.12:g.160412632=
JAZF1	**rs10486567**	G/A/C	G	GRCh38.p13 chr 7	NC_000007.14:g.27936944=
LMTK2	**rs6465657**	C/T	C	GRCh38.p13 chr 7	NC_000007.14:g.98187015=
SLC25A37	**rs2928679**	A/G/T	A	GRCh38.p13 chr 8	NC_000008.11:g.23581462=
NKX3.1	**rs1512268**	T/A/G	T	GRCh38.p13 chr 8	NC_000008.11:g.23668950=
CTBP2	**rs4962416**	T/C	T	GRCh38.p13 chr 10	NC_000010.11:g.125008303=
MSMB	**rs10993994**	C/A/T	C	GRCh38.p13 chr 10	NC_000010.11:g.46046326=
HNF1B	**rs4430796**	A/C/G/T	A	GRCh38.p13 chr 17	NC_000017.11:g.37738049=
HNF1B	**rs11649743**	A/G	A	GRCh38.p13 chr 17	NC_000017.11:g.37714971=
TMPRSS2:<br>ERG	**rs16901979**	C/A	C	GRCh38.p13 chr 8	NC_000008.11:g.127112671=
CASC17	**rs1859962**	G/C/T	G	GRCh38.p13 chr 17	NC_000017.11:g.71112612=
LOC1019280<br>59	**rs34579442**	TTTTTTT<br>TTTTTTT<br>TT/TTTTT<br>TTTTTTT<br>T/TTTTTT<br>TTTTTTT<br>T/TTTT...	TTTTTTTTTT<br>TTTTTT	GRCh38.p13 chr 1	NC_000001.11:g.153927425_15<br>3927440=
MEIS1-AS3	**rs74702681**	C/T	C	GRCh38.p13 chr 2	NC_000002.12:g.66425753=
BCL2L11	**rs11691517**	T/G	T	GRCh38.p13 chr 2	NC_000002.12:g.111135519=
CDCA7	**rs34925593**	T/A/C	T	GRCh38.p13 chr 2	NC_000002.12:g.173369819=
CASP8	**rs59308963**	ATTCTGT<br>CATTCTG<br>TC/ATTC<br>TGTC	ATTCTGTCA<br>TTCTGTC/AT<br>TCTGTC	GRCh38.p13 chr 2	NC_000002.12:g.201258757_20<br>1258772=
DUBR	**rs1283104**	C/G	C	GRCh38.p13 chr 3	NC_000003.12:g.107243674=
MBNL1	**rs182314334**	T/C	T	GRCh38.p13 chr 3	NC_000003.12:g.152286413=
RNU6–456P	**rs10793821**	C/A/T	*C*	GRCh38.p13 chr 5	NC_000005.10:g.134500518=
DOCK2	**rs76551843**	A/G	A	GRCh38.p13 chr 5	NC_000005.10:g.169745129=

COL23A1	**rs4976790**	G/A/C/T	G	GRCh38.p13 chr 5	NC_000005.10:g.178541914=
ATAT1	**rs12665339**	A/G	A	GRCh38.p13 chr 6	NC_000006.12:g.30633455=
UHRF1BP1	**rs9469899**	G/A	G	GRCh38.p13 chr 6	NC_000006.12:g.34825347=
MAD1L1	**rs527510716**	G/C	G	GRCh38.p13 chr 7	NC_000007.14:g.1904901=
ITGB8	**rs11452686**	AAAAAAAAAAAAAAA/AAAAAAAAA/AAAAAAAAAA/AAAAAAAAA...	AAAAAAAAAAAAAAA	GRCh38.p13 chr 7	NC_000007.14:g.20374488_20374502=
SUGCT	**rs17621345**	A/C	A	GRCh38.p13 chr 7	NC_000007.14:g.40835593=
HAUS6	**rs1048169**	T/C/G	T	GRCh38.p13 chr 9	NC_000009.12:g.19055967=
TOR1A	**rs1182**	C/A	C	GRCh38.p13 chr 9	NC_000009.12:g.129813781=
RNLS	**rs1935581**	C/A/T	C	GRCh38.p13 chr 10	NC_000010.11:g.88435392=
TCF7L2	**rs7094871**	C/G/T	C	GRCh38.p13 chr 10	NC_000010.11:g.112952395=
INCENP	**rs2277283**	T/A/C/G	T	GRCh38.p13 chr 11	NC_000011.10:g.62140968=
KDM2A	**rs12785905**	G/C	G	GRCh38.p13 chr 11	NC_000011.10:g.67184494=
EMSY	**rs11290954**	C/-	C	GRCh38.p13 chr 11	NC_000011.10:g.76549500=
B3GAT1	**rs878987**	A/G	A	GRCh38.p13 chr 11	NC_000011.10:g.134396478=
FBRSL1	**rs7295014**	G/A	G	GRCh38.p13 chr 12	NC_000012.12:g.132491403=
MMP14	**rs1004030**	T/C	T	GRCh38.p13 chr 14	NC_000014.9:g.22836440=
PAX9	**rs11629412**	G/A/C	G	GRCh38.p13 chr 14	NC_000014.9:g.36669089=
RFX7	**rs33984059**	A/G	A	GRCh38.p13 chr 15	NC_000015.10:g.56093670=
CHD3	**rs28441558**	T/C	T	GRCh38.p13 chr 17	NC_000017.11:g.7899800=
RNF43	**rs2680708**	G/A	G	GRCh38.p13 chr 17	NC_000017.11:g.58378759=
TCF4	**rs28607662**	T/C	T	GRCh38.p13 chr 18	NC_000018.10:g.55563628=
MYO9B	**rs11666569**	C / T	C	GRCh38.p13 chr 19	NC_000019.10:g.17103263=
POU2F2	**rs61088131**	T/C	T	GRCh38.p13 chr 19	NC_000019.10:g.42196795=
ARHGAP6	**rs17321482**	C/T	C	GRCh38.p13 chr X	NC_000023.11:g.11464514=
PCAT19	**rs11672691**	G/A	G	GRCh38.p13 chr 19	NC_000019.10:g.41479679=
MLH3	**rs28756990**	C/A/T	C	GRCh38.p13 chr 14	NC_000014.9:g.75047435=
OR2A5	**rs2961144**	A/G	A	GRCh38.p13 chr 7	NC_000007.14:g.144050777=
ESR2	**rs58262369**	C/T	C	GRCh38.p13 chr 14	NC_000014.9:g.64227194=
ATM	**rs1800057**	C/A/G	C	GRCh38.p13 chr 11	NC_000011.10:g.108272729=
CDKN1B	**rs2066827**	T/A/C/G	T	GRCh38.p13 chr 12	NC_000012.12:g.12718165=

KLK3	**rs2735839**	A/C/G/T	A	GRCh38.p13 chr 19	NC_000019.10:g.50861367=
PPFIBP2	**rs12791447**	A/C/G	A	GRCh38.p13 chr 11	NC_000011.10:g.7535346=
PCAT2	**rs114798100**	A/G	A	GRCh38.p13 chr 8	NC_000008.11:g.127073189=
PRNCR1	**rs72725879**	C/T	C	GRCh38.p13 chr 8	NC_000008.11:g.127091724=
TP63	**rs56197129**	C/A	C	GRCh38.p13 chr 3	NC_000003.12:g.189817054=
TP63	**rs6782221**	G/A/C	G	GRCh38.p13 chr 3	NC_000003.12:g.189819367=
TP63	**rs6795465**	T/C	T	GRCh38.p13 chr 3	NC_000003.12:g.189819732=
TP63	**rs56413159**	G/C	G	GRCh38.p13 chr 3	NC_000003.12:g.189819820=
TP63	**rs73199732**	C/G	C	GRCh38.p13 chr 3	NC_000003.12:g.189820451=
TP63	**rs55851920**	T/C	T	GRCh38.p13 chr 3	NC_000003.12:g.189820771=
TP63	**rs7616437**	A/G	A	GRCh38.p13 chr 3	NC_000003.12:g.189833631=
WNT1	**rs855723**	G/A/C/T	G	GRCh38.p13 chr 12	NC_000012.12:g.48976764=
EGFR	**rs2072454**	C/T	C	GRCh38.p13 chr 7	NC_000007.14:g.55146655=
EGFR	**rs2270247**	G/T	G	GRCh38.p13 chr 7	NC_000007.14:g.55146954=
EGFR	**rs730437**	A/C	A	GRCh38.p13 chr 7	NC_000007.14:g.55147325=
EGFR	**rs2075109**	T/C	C	GRCh38.p13 chr 7	NC_000007.14:g.55151210=
EGFR	**rs2075110**	C/G/T	T	GRCh38.p13 chr 7	NC_000007.14:g.55151466=
EGFR	**rs6944695**	T/A/C	T	GRCh38.p13 chr 7	NC_000007.14:g.55151597=
EGFR	**rs2075111**	C/A/G/T	G	GRCh38.p13 chr 7	NC_000007.14:g.55151614=
EGFR	**rs12718946**	C/G	G	GRCh38.p13 chr 7	NC_000007.14:g.55153754=
Next TNRC6B	**rs9632117**	C/A		GRCh38.p13 chr 4	NC_000004.12:g.49493951=
Next RAD23 B and KLF4	**rs817826**	C/T	C	GRCh38.p13 chr 9	NC_000009.12:g.107394019=
Next MLPH	**rs7584330**	A/G	G	GRCh38.p13 chr 2	NC_000002.12:g.237478585=
VDR	**rs7975232**	C/A	C	GRCh38.p13 chr 12	NC_000012.12:g.47845054=
CCAT2	**rs6983267**	G/T	G	GRCh38.p13 chr 8	NC_000008.11:g.127401060=
CASC8	**rs7000448**	C / T	T	GRCh38.p13 chr 8	NC_000008.11:g.127428925=
PCAT1	**rs6983561**	A / C	C	GRCh38.p13 chr 8	NC_000008.11:g.127094635=
LOC1001312 41	**rs6501455**	A/G/T	A	GRCh38.p13 chr 17	NC_000017.11:g.71205670=
